# Interaction and dynamic changes of microbial communities and volatile flavor compounds during the fermentation process of coffee flower rice wine

**DOI:** 10.3389/fmicb.2024.1476091

**Published:** 2024-09-19

**Authors:** Kunyi Liu, Rui Su, Qi Wang, Xiaojing Shen, Bin Jiang, Liran Yang, Zelin Li, Jia Zheng, Pingping Li

**Affiliations:** ^1^School of Wuliangye Technology and Food Engineering and School of Modern Agriculture, Yibin Vocational and Technical College, Yibin, China; ^2^Sericulture and Apiculture Research Institute, Yunnan Academy of Agricultural Sciences, Mengzi, China; ^3^College of Science, Yunnan Agricultural University, Kunming, China; ^4^Agro-Products Processing Research Institute, Yunnan Academy of Agricultural Sciences, Kunming, China; ^5^Wuliangye Yibin Co., Ltd., Yibin, China

**Keywords:** microbial community, volatile flavor compound, sensory evaluation, coffee flower rice wine, interaction

## Abstract

To develop a unique flavor of rice wine, coffee flowers (by-products of the coffee industry) were added because of their biologically active compounds that are conducive to health, and the fermentation parameters were optimized. In addition, the dynamic changes of microbial communities and volatile flavor compounds (VFCs) during the different fermentation stages were investigated. After the optimization of the fermentation parameters, a novel product, i.e., the coffee flower rice wine (CFRW), was obtained with a bright yellow transparent, fragrant, and harmonious aroma and mellow and refreshing taste by sensory evaluation, when 4.62% of the coffee flowers and 1.93% koji were added and fermented at 24.10°C for 3.88 days. The results showed that *Lactococcus* was the dominant bacteria, accounting for 87.0–95.7%, while *Rhizopus* and *Cladosporium* were the main fungi, accounting for 68.2% and 11.3% on average, respectively, in the fermentation process of the CFRW. Meanwhile, twenty-three VFCs were detected in the CFRW, which included three alcohols, six terpenes, ten esters, three aromatics, and one furan. The correlation analysis revealed that there were 16 significant positive correlations and 23 significant negative correlations between the bacterium and VFCs (|*ρ*| > 0.6, *p* < 0.05), while there were 12 significant positive correlations and one significant negative correlation between the fungi and VFCs (|ρ| > 0.6, *p* < 0.05). Furthermore, five VFCs, including linalool, geraniol, ethyl acetate, 1-hexanol, and 3-methyl-1-butanol, contributed vital flavors to the CFRW, and they were all significantly negatively correlated with the changes of *Massilia* and *Acinetobacter* (|*ρ*| > 0.6, *p* < 0.05). Moreover a significant positive correlation was found between the relative abundance of *Lactococcus* and the contents of 3-methyl-1-butanol and ethyl acetate (|ρ| > 0.6, *p* < 0.05). Therefore, this study provides a valuable theoretical basis for further improving the quality and production technology of CFRW.

## 1 Introduction

Chinese rice wine is a traditional alcoholic beverage, with a history of more than 9,000 years, which gives it the reputation of being one of the oldest brewed wines in the world (Jiao et al., 2017; Mao et al., 2023). Undoubtedly, it has great popularity among consumers in China and East Asia due to its full aroma, low alcoholic content, unique flavor, and potential value in the medical field (Zhao et al., 2018; Chen et al., 2021; Mao et al., 2023). Numerous studies have reported that Chinese rice wine contains a wide range of chemical compounds, including carbohydrates, proteins, organic acids, vitamins, minerals, and rich bioactive substances (Jung et al., 2014; Lu et al., 2017; Hipol and Alma-in, 2018). Moreover, Chinese rice wine exhibits antioxidant properties that can remove free radicals and protect cells from oxidation, which is beneficial to the health of consumers (Que et al., 2006; Chay et al., 2020; Shen et al., 2021).

The flavor and aroma of rice wine are important indicators that are well-known for evaluating wine quality and determining consumer preference. Microorganisms produce alcohol and other metabolites during the fermentation process, wherein flavor and aroma are formed (Chen et al., 2018; Ren et al., 2019). Hence, microorganisms play a crucial role in the unique flavor formation of rice wine. Over the years, according to available document data, researchers have found that they are responsible for different contributions to the final product (Jiang et al., 2020). Furthermore, it is important to note that the raw materials used for fermented rice wine may also play an important role in its flavor and taste. Hitherto, many scholars have focused on the microbial dynamics and flavor changes in the fermentation broth of rice wine from different regions (Chen et al., 2021). Nevertheless, rice wine with a high quality and unique taste has gradually attracted the attention of researchers and has been favored by consumers as a result of the improvement in living conditions in recent years. The rice wine made with traditional raw materials can no longer fully meet the needs of current consumers. Therefore, new auxiliary foodstuffs should be applied to the traditional brewing system of rice wine to improve the commercial value and develop products with a more unique taste and flavor.

Coffee is a principal industry in Yunnan, China. However, a large number of coffee flowers fall to the ground every year and are not fully utilized, which causes abundant waste in the coffee industry (Nguyen et al., 2019). These coffee flowers can be harvested after pollination and reused in the bioenergy development and can be prepared as important ingredients in foods, beverages, and cosmetics as they contain vitamins, sugars, and rich bioactive compounds that possess potential benefits for human health because of their antioxidant, anti-fibrosis, and antiviral activities and help prevent cardiovascular diseases, cancers, and diabetes (Mlcek and Rop, 2011; Mak et al., 2013; Tseng et al., 2000; Lachenmeier et al., 2021). Recently, it has been proposed that coffee flowers can be used as a promising source in the production of beverages, bio-sugars, and functional healthy foods (Wirz et al., 2022). Interestingly, coffee flowers contain flavor compounds such as methyl jasmonate, phenylethanol, geraniol, and indole, which can emit a highly pleasant jasmine-like floral aroma (Emura et al., 1997; Stashenko et al., 2013; Shen et al., 2023). Therefore, coffee flowers can be used as raw materials to improve the flavor of food.

Hence, if coffee flower as an innovative material is added to the traditional fermentation process, we may be able to develop a new rice wine product with a unique taste and potential medical benefits, which can be of great significance to improve the additional value of coffee flowers and meet the personalized demands of consumers. However, the effects of coffee flowers on rice wine’s flavor profile and microbial community remain unclear. In this study, we will evaluate the sensory characteristics, volatile flavor compounds (VFCs), and their correlation with the microbial communities involved in the fermentation of coffee flower rice wine (CFRW) under optimal fermentation parameters, thus providing a theoretical basis for its large-scale production.

## 2 Materials and methods

### 2.1 Chemicals and reagents

Coffee flowers were harvested and collected from Baoshan, Yunnan, China in May 2023, and glutinous rice (the starch content was 78.34%) was obtained from Shenyang Xinchang Grain Trading Co., Ltd. (Liaoning, China). Koji (rice flour fermented using *Rhizopus oryzae*) was purchased from Angel Yeast Co., Ltd. (Hubei, China). Reagents 3-methyl-1-butyl-1,1-d2 alcohol, n-hexyl-1,1-d2 alcohol, (±)-linalool-d3 (vinyl-d3), ethyl acetate-d3, ethyl hexanoate-d11, ethyl octanoate-d15, 2-phenylethyl acetate-d3, and ethyl octanoate-d15 were purchased from Chengdu Manst Biotechnology Co., Ltd. (Chengdu, China).

### 2.2 Test flow of the coffee flower rice wine

**Figure 1:**
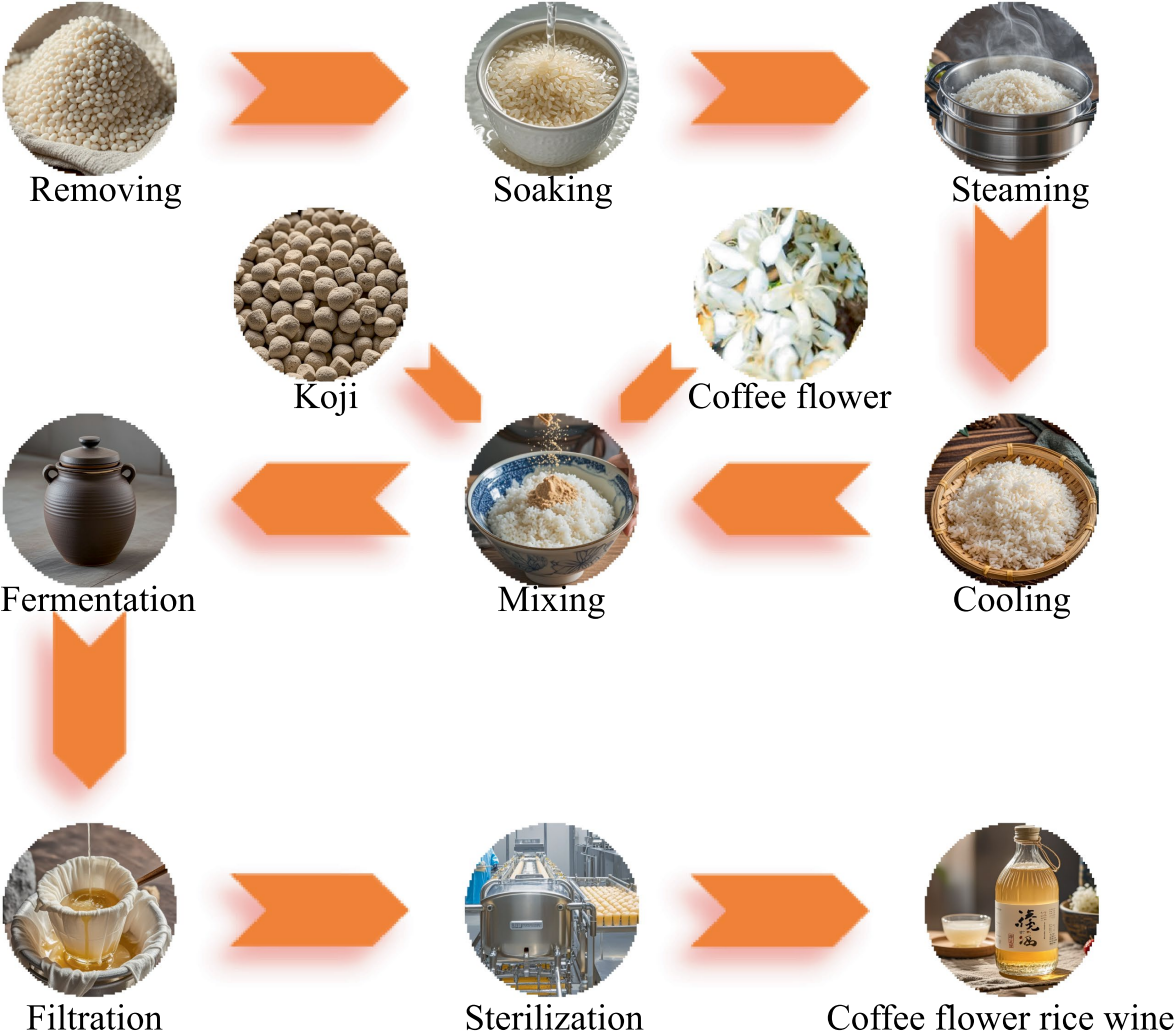
Test flow of the coffee flower rice wine.

CFRW was fermented using traditional glutinous rice and coffee flowers as feedstock. The brewing test flow was performed as follows: the glutinous rice was washed to remove impurities and soaked in a diploid amount of pure water for 24 h and then drained; thereafter, the soaked glutinous rice was steamed for 45 min, followed by cooling to 30°C. According to the experimental design, a certain amount of the koji and fresh coffee flowers (cleaned with aseptic water beforehand) were added to the rice grains in different proportions; meanwhile, they were evenly mixed by stirring with hands and then put into a jar for static fermentation. It must be noted that the mixture was stacked from the edge of the jar to the center. When one-third of the fermentation raw materials remained, they were only added to the edge of the fermentation jar so that the middle was lower than the edge; after the fermentation, the liquor was pressed with a filter gauze and boiled at 65°C for 30 min to obtain the final product (Figure 1). The fermented samples were harvested every day and conserved at −80°C until required. During the different fermentation stages of 3–7 days, three parallel samples were collected each time.

### 2.3 Single-factor and response surface experiments

The single-factor test was performed to evaluate the addition of the coffee flowers (0, 2, 4, 6, 8, and 10%), the addition of the koji (0.5, 1.0, 1.5, and 2.0%), the fermentation temperature (22, 24, 26, 28, and 30°C), and the fermentation time (3, 4, 5, 6, and 7 days) on the sensory scores of the CFRW. One factor was kept constant, and the others were changed for each single-factor test. Each group of experiments was repeated three times for a single factor. The Box–Behnken design was employed to optimize the brewing process of the CFRW further and evaluate the effects of the interactions on the quality of the CFRW based on the results of the single-factor experiments. In total, a 29-trial design was generated, and the center points per block were five in this study. The four independent variables were the addition of the coffee flowers (2, 4, and 6%), the addition of the koji (1.5, 2.0, and 2.5%), the fermentation temperature (22, 24, and 26°C), and the fermentation time (3, 4, and 5 days). Each variable corresponded to three coded levels: low (−1), middle (0), and high (+1). Then, three parallel trials were conducted to verify the reliability of the above optimal fermentation parameters of the CFRW determined by the response surface experiments.

### 2.4 Sensory evaluation of the CFRW

**Figure 2:**
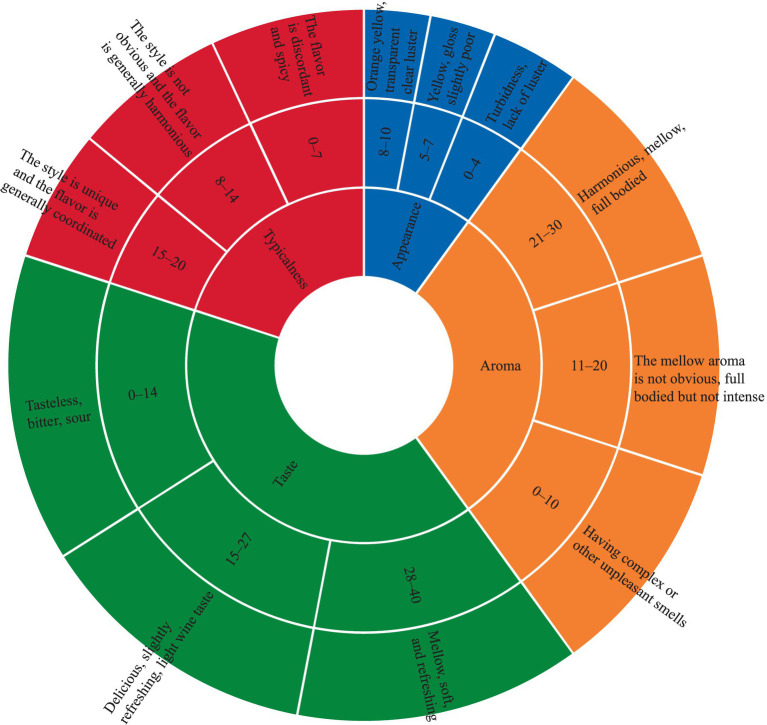
Sensory evaluation standards of the coffee flower rice wine.

A quantitative characterization of the sensory properties of the CFRW samples was conducted with official methods of GB/T 13662—2018. A training panel of 11 judges was organized to conduct a sensory evaluation on all indicators of the CFRW product using a sensory rating scale. The panel consisted of 11 members, including five men and six women, and they aged from 20 to 30 years old. Each member had more than 3 years of wine-tasting and sensory analysis experience. A total of four sensory descriptors were considered, and the corresponding scoring standards are shown in Figure 2. The comprehensive score of all indicators was taken as the total score. Meanwhile, the members began by evaluating the aroma profile characteristics of the CFRW samples and selecting six common quality descriptors (floral fragrance, fruity fragrance, rice fragrance, honey fragrance, nutty fragrance, and creamy fragrance) that best represented the CFRW samples. The intensities of the aroma attributes were scored on a scale ranging from 0 to 10; the higher the score, the stronger the intensity.

### 2.5 Microbiological analyses

#### 2.5.1 Sample preparation

A microbial community analysis of the CFRW was performed under the optimal fermentation parameters. The CFRW samples with different fermentation times (0, 1, 2, 3, and 4 days) were collected and labeled as F0, F1, F2, F3, and F4, respectively. Each sample was repeated three times. Then, they were put in sterile polyethylene bags and stored at −80°C in a freezer until further analysis. The microbial DNA was extracted using the HiPure Soil DNA Kits (D3142, Guangzhou American Biotechnology Co., Ltd., Guangzhou, China) according to the protocols of the manufacturer. The final DNA quality was determined using a NanoDrop 2000 microspectrophotometer (Thermo Fisher Scientific, United States) and agarose gel electrophoresis. The primers with a barcode used for amplifying the V3–V4 region of the bacteria 16S rDNA were 341F and 806R. The primers applied to amplify the ITS2 region of the fungi gene were ITS3 and ITS4. Polymerase chain reaction (PCR) procedures were carried out by referring to previously reported methods (Liu et al., 2022; Luan et al., 2018). Amplicons were extracted from the agarose gels and purified using the AxyPrep DNA Gel Extraction Kit (Axygen Biosciences, Union City, CA, United States) according to the standard protocols. Then, the PCR product was quantified using the ABI StepOnePlus Real-Time PCR System (Life Technologies, Foster City, United States) and was pooled in equal amounts. After that, sequencing of the amplicon library was performed on the Illumina Novaseq 6,000 platform at Guangzhou Gene Denovo Co., Ltd. (Guangzhou, China). The raw reads were deposited into the National Center for Biotechnology Information (NCBI) Sequence Read Archive (SRA) database (Accession Number: PRJNA869838 for bacterial and PRJNA869566 for fungal).

#### 2.5.2 Bioinformatics analysis of the sequencing data

The raw sequencing data were filtered and analyzed using FLASH software (v1.2.7) and QIIME software (v1.8.0). A clustering analysis of the operational taxonomic unit (OTU) was conducted using USEARCH software (v10.0). The OTU sequences of the bacterial and fungal genes were annotated using the SILVA/16S rDNA database and the UNITE database, respectively (Qian et al., 2023).

### 2.6 Determination of the VFCs

5.0 mL of the CFRW sample and 10 μL internal standards of isotope (100 μg/g 3-methyl-1-butyl-1,1-d2 alcohol, n-hexyl-1,1-d2 alcohol, (±)-linalool-d3, ethyl acetate-d3, ethyl hexanoate-d11, ethyl octanoate-d15, 2-phenylethyl acetate-d3, and ethyl octanoate-d15) were fully mixed and placed in headspace glass vials for balancing for 15 min under 60°C and 250 r/min. Then, the 0.5 mL balanced sample was collected with a 2.5 mL airtight syringe, which was then pre-heated to 80°C and placed in the headspace vials. The VFCs were extracted for 45 min at 60°C using a divinylbenzene/carboxen/polydimethylsiloxane (DVB/CAR/PDMS) fiber and desorbed at the GC injection port at 250°C for 3 min for GC–MS analysis. Each sample was repeated three times. Subsequent GC–MS analysis of the VFCs was performed according to the method reported in the literature (Yang et al., 2022). The VFCs of the CFRW were identified by matching the mass spectral databases (NIST 1.6 and Wiley 6.0), and their relative contents were calculated according to the internal standard method.

The odor activity value (OAV) of the VFCs in the CFRW was defined as the ratio of the concentration of a volatile compound to its odor detection threshold, which was found in the literature (Ferreira et al., 2002; Gomez-Miguez et al., 2007; Rahayu et al., 2017). Only compounds with an OAV > 1 can be considered contributing to the aroma of CFRW (Zhu et al., 2021).

### 2.7 Statistical analysis

The data of response surface methodology (RSM) were analyzed using Design-Expert 10.0.1.0 software. The data of the sensory scores and their differences among the groups were analyzed by one-way ANOVA using SPSS 22.0 software (IBM, Chicago, IL, United States). All samples were subjected to three biological replicates. The data were presented in the form of averages and standard deviations. Spearman correlation analysis was performed to assess the relationship between the dominant genera and volatile metabolites using R Project 3.6.1. The stacked bar plot of the microbial community composition was visualized in the R project ggplot2 package (version 2.2.1).

## 3 Results

### 3.1 Optimization of the fermentation parameters of the CFRW

#### 3.1.1 Single-factor experiment results

**Figure 3:**
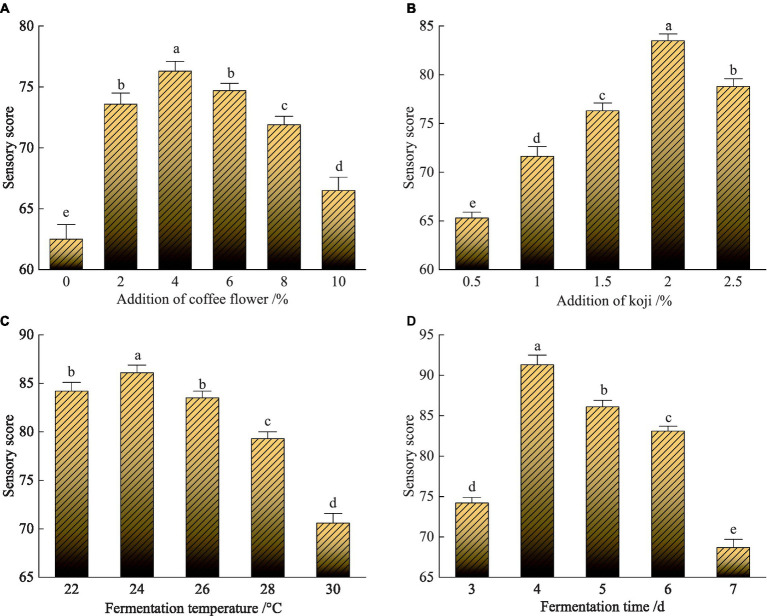
Effect of the addition of the coffee flowers on the sensory scores of the coffee flower rice wine (CFRW) **(A)**, effect of the addition of the koji on the sensory scores of the CFRW **(B)**, effect of the fermentation temperature on the sensory scores of the CFRW **(C)**, effect of the fermentation time on the sensory scores of the CFRW **(D)**.

Figure 3A shows the effect of the addition of the coffee flowers on the sensory score of the CFRW. Overall, the addition of the coffee flowers had a significant effect on the sensory score of the CFRW. As more coffee flowers were added, the sensory score of the CFRW presented a trend where they first rose and then fell. It reached the highest score (76.3), and it was significantly higher than the others (*p* < 0.05) when 4% of the coffee flowers were added. However, sequentially appending more coffee flowers resulted in a significant decrease in the sensory score of the CFRW (*p* < 0.05). In particular, it got the lowest sensory score of 66.5 when 10% of the coffee flowers were added to the CFRW. Thus, 4% was regarded as the optimum addition of the coffee flowers.

It can be seen that the sensory score of the CFRW continuously increased from 65.3 to 83.5, with the increase in the addition of the koji from 0.5% to 2.0% (Figure 3B). However, the sensory score dropped significantly to 78.8 when the amount of koji was 2.5%.

Overall, the sensory score of the CFRW descended with the ascending fermentation temperature from 24 to 30°C (Figure 3C). In addition, the sensory score of the CFRW brewed at 26°C was slightly lower (83.5) than that brewed at 22°C, but the difference was not significant. In the fermentation process, the excessive temperature significantly reduced the sensory score of the CFRW (*p* < 0.05). When the temperature was 24°C, the fermented CFRW had the highest sensory score of 86.1, which was significantly higher than the others (*p* < 0.05). However, when it was higher than the appropriate temperature, especially at 30°C, the CFRW was subjected to the lowest score of 70.6.

As shown in Figure 3D, the fermentation time markedly affected the sensory scores of the CFRW. It first increased and then decreased with the extension of the fermentation time. When fermented for 4 days, the sensory score of the CFRW was as high as 91.3, which was significantly higher than the others (*p* < 0.05). However, when the fermentation time was continued to extend, the sensory score of the CFRW decreased significantly (*p* < 0.05). In particular, it achieved the lowest sensory score (68.7) when it was fermented for 7 days.

#### 3.1.2 The optimal fermentation parameters and sensory evaluation of the CFRW

**Figure 4:**
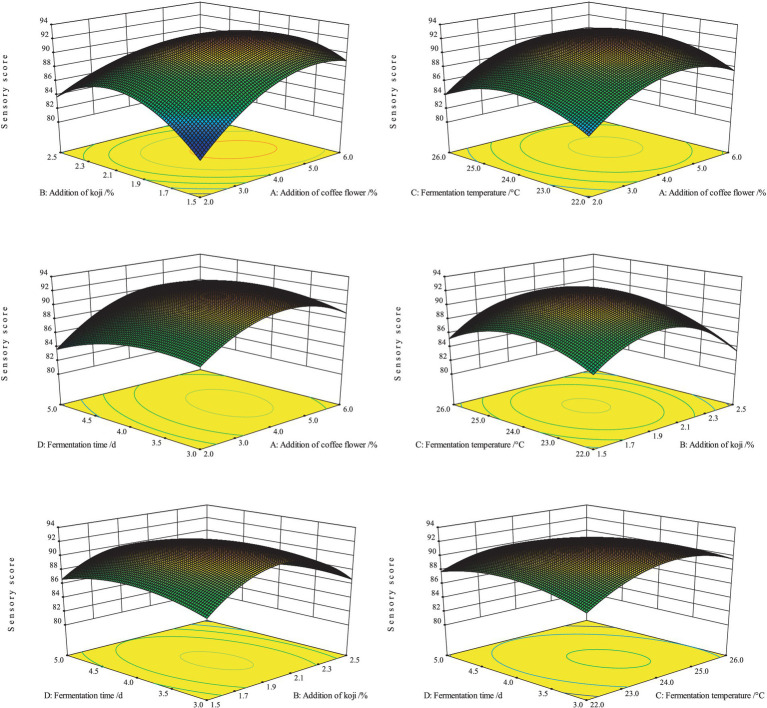
Response surface of the interaction effects on the sensory scores of the coffee flower rice wine.

A response surface test was performed to determine the optimal fermentation parameters of the CFRW. Moreover, a sensory evaluation was conducted to evaluate the effect of different fermentation parameters on the sensory performance of the CFRW. According to the experimental design, 29 trials were conducted, and the corresponding result of the ANOVA is illustrated in Table 1. As shown in Table 1, the model was extremely significant (*p* < 0.01) but not significant (*p* > 0.05) for the lack of fit, which implied that the regression equation was well fitted. The four factors had different contributions to the response value of the CFRW. Among these factors, the addition of the coffee flowers had the most significant effect on the sensory score of the CFRW, followed by the addition of the koji and the fermentation time. In this case, the line term *A* and the quadric terms *A*^2^, *B*^2^, and *C*^2^ had very significant effects (*p* < 0.01) on the sensory score of the CFRW. However, among the interaction terms, the only significant interaction (*p* < 0.05) effect was found in the term *AB* (Table 1). As shown in Figure 4, the response surface formed by the factors *A* and *B* had the steepest slope, which verified that the interaction term *AB* had a significant impact on the sensory evaluation of the CFRW (*p* < 0.05). The final second-order quadratic regression equation of the response was evaluated as follows:


$$\begin{array}{l} Y=92.2+1.93A-0.69B+0.2C-0.69D-2.35\mathrm{AB}-\\ \;0.025\;\mathrm{AC}+0.68\;\mathrm{AD}+0.27\;BC-0.9BD-1.1CD-\\ \;3.51A^{2}-4.68B^{2}-2.89C^{2}-1.7D^{2} \end{array}$$

**Table 1:** Variance analysis on the sensory evaluation of the coffee flower rice wine.

Source of variation	Sum of squares	Degree of freedom	Mean square	*F*-value	*p*-value	Significance
Model	294.91	14	21.06	4.78	0.003	**
*A*	44.85	1	44.85	10.17	0.0066	**
*B*	5.74	1	5.74	1.30	0.2730	
*C*	0.48	1	0.48	0.11	0.7463	
*D*	5.74	1	5.74	1.30	0.2730	
*AB*	22.09	1	22.09	5.01	0.0420	*
*AC*	0.0025	1	0.0025	0.0005	0.9813	
*AD*	1.82	1	1.82	0.41	0.5307	
*BC*	0.30	1	0.30	0.07	0.7972	
*BD*	3.24	1	3.24	0.73	0.4058	
*CD*	4.84	1	4.84	1.10	0.3125	
*A* ^2^	80.03	1	80.03	18.15	0.0008	**
*B* ^2^	141.77	1	141.77	32.15	< 0.0001	**
*C* ^2^	54.08	1	54.08	12.27	0.0035	**
*D* ^2^	18.75	1	18.75	4.25	0.0583	
Residual	61.73	14	4.41			
Lack of fit	52.15	10	5.21	2.18	0.2358	
Pure error	9.58	4	2.39			
Cor total	356.63	28				

“*” indicates a significant difference at the 0.05 level, and “**” indicates an extremely significant difference at the 0.01 level.

In the above formula, *Y* represents the sensory score. *A*, *B*, *C,* and *D* are the independent variables of the addition of the coffee flowers (%), the addition of the koji (%), the fermentation temperature (°C), and the fermentation time (d), respectively. Based on the present study, the CFRW with the highest theoretical sensory score (92.59) may be obtained if produced under 4.62% coffee flowers and 1.93% koji fermenting at 24.1°C for 3.88 days. To verify the reliability of the response surface test results, three parallel experiments were carried out. Considering the operability of the experiment, the optimal production technology obtained by the software was appropriately adjusted to the addition of 4.6% coffee flowers and 1.9% koji, fermented at 24.1°C for 4 days. After that, the sensory evaluation was performed on the CFRW products fermented with the optimal fermentation parameters. Their average sensory score was 92.8 ± 0.7, which was stable with a deviation of <1.0% and thus proved that the result was reasonable and reliable. These samples had an orange-yellow and transparent color, rich and harmonious aroma, and mellow and refreshing taste. Overall, a unique style of coffee flower rice wine was obtained.

### 3.2 Microbial community analysis of the CFRW

#### 3.2.1 The bacterial diversity during the different fermentation stages

The dynamics variability in the bacterial community of the CFRW was analyzed during different fermentation stages under optimal conditions based on the RSM. A total of 551,934 sequences of the bacterial communities were acquired, which were passed through the quality control filters, and the chimera was removed. A series of alpha diversity indices were estimated to determine the intra-and inter-variability in the bacterial community among the five groups. As shown in Supplementary Figure S1, the Shannon index and Simpson index indicated that the bacterial community diversity was highest before the fermentation of the CFRW (F0), whereas it dramatically decreased after the fermentation. Among the different fermentation stages, the bacterial diversity underwent a process of increasing first and then decreasing.

**Figure 5:**
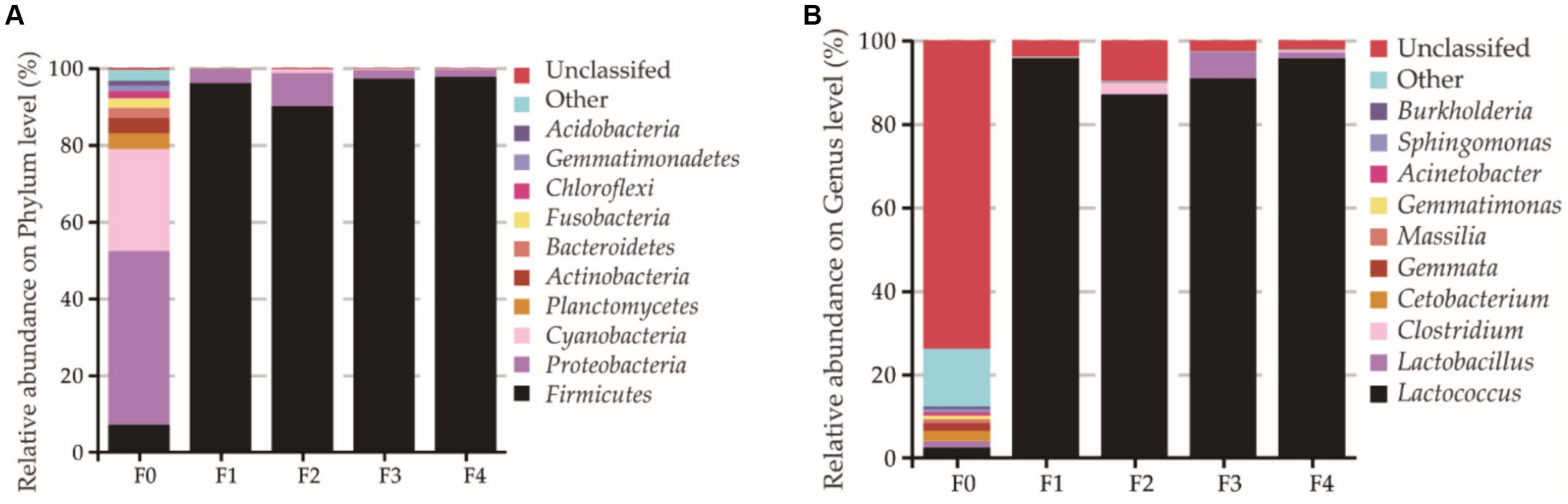
Bacterial community distribution of the different fermentation stages’ samples of the coffee flower rice wine (CFRW). Relative abundances of the most abundant bacterium at the phylum **(A)** and genus **(B)** were analyzed. F0 stands for the day 0 sample, and the remainder is deduced by analogy. Unclassified stands for unknown bacterium.

The 16S rRNA gene sequences indicated that 197 bacterial genera were found in the fermentation broth, which were classified into 27 phyla and 134 families. Most bacteria of the CFRW belonged to Firmicutes during the fermentation, which accounted for 95.2% of the bacteria (Figure 5A). As shown in Figure 5B, the relative abundance of the unclassified bacterium genus appeared at its maximum (73.9%) on day 0 but then sharply decreased at the beginning of the fermentation. Interestingly, the relative abundance presented a converse trend for *Lactococcus*, which started increasing rapidly from the 1^st^ day of the fermentation, reaching the highest level on the last day, which ranged from 87.0 to 95.7%. Among all groups, *Lactococcus* was probably the main bacterium genus, but beyond that, a small proportion of *Lactobacillus* (6.1%) was also detected on the 3^rd^ day.

#### 3.2.2 The fungal diversity during the different fermentation stages

**Figure 6:**
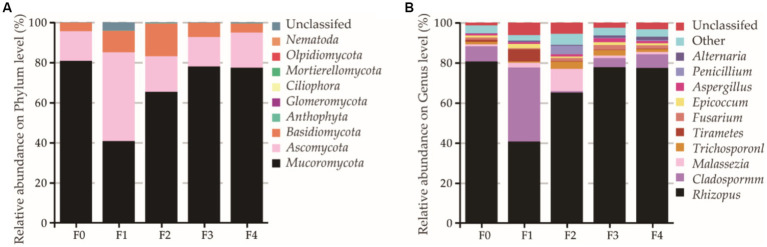
Fungal community distribution of the different fermentation stages’ samples of the coffee flower rice wine (CFRW). Relative abundances of the most abundant fungus at the phylum **(A)** and genus **(B)** were analyzed. F0 stands for the day 0 sample, and the remainder is deduced by analogy. Unclassified stands for unknown fungus.

As the fermentation proceeded, the fungal diversity increased first but then decreased in the later stages of the fermentation (Supplementary Figure S2). A total of 3,468,418 effective fungal sequences were obtained from the fermentation broth of the CFRW after quality control processing. After that, they were counted and then annotated into 9 phyla, 76 families, and 102 genera. Most fungal sequences of the CFRW belonged to Mucoromycota, Ascomycota, and Basidiomycota, which accounted for 68.4, 21.8, and 8.6% of the fungal sequences, respectively (Figure 6A). Undoubtedly, *Rhizopus* was dominant on day 0 of the broth, which had not yet fermented (Figure 6B). However, it reduced abruptly on the 1^st^ day of the fermentation and markedly increased and eventually tended to be stable on the 4^th^ day. Except for *Rhizopus*, *Cladosporium* had a higher relative proportion than the other fungus genera on the first and the last 2 days of the fermentation. The relative abundance of *Malassezia* on the 2^nd^ day was evidently higher than that on other days. The statistical analysis showed that there was no significant difference (*p* > 0.05) in the fungal community diversity among all groups, but it peaked on the 2^nd^ day of the fermentation and then reduced with mild fluctuations.

### 3.3 Volatile flavor compounds analysis

**Figure 7:**
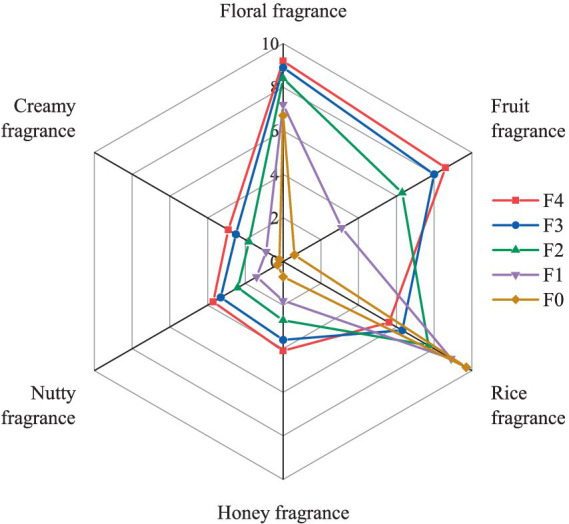
Aroma profile characteristics of the coffee flower rice wine during the fermentation process.

A total of 23 volatile flavor compounds (VFCs) were successfully detected and identified in the fermented samples of the CFRW, which included three alcohols, six terpenoids, ten esters, three aromatics, and one furan. The details of the result are presented in Table 2. The concentrations of almost all VFCs were significantly increased by the fermentation (*p* < 0.05). When it was not fermented, 3-methyl-1-butanol and geraniol were the main VFCs of the CFRW, and their concentrations were 1349.72 ± 163.33 and 637.35 ± 79.30 mg/kg, respectively, while the concentrations of most of the remaining VFCs were very low. In the brewing process of the CFRW, the concentration of 3-methyl-1-butanol significantly increased (*p* < 0.05), which was the predominant component (2072.60 ± 67.51 mg/kg) on the 1^st^ day of the fermentation, followed by geraniol, 1-hexanol, ethyl acetate, and 2-methyl-1-propanol. After 2–4 days of the fermentation, the main VFCs in the CFRW were geraniol, 3-methyl-1-butanol, ethyl acetate, 1-hexanol, and *β*-pinene. On the 4^th^ day, the concentrations of 3-methyl-1-butanol and 1-hexanol were significantly higher than those on other fermentation stages (*p* < 0.05), reaching a peak of 1406.52 ± 74.85 and 3152.65 ± 80.73 mg/kg, respectively. It is worth noting that the concentration of phenylethyl alcohol significantly increased in the later fermentation stage of the CFRW (*p* < 0.05) and achieved the highest level of 405.40 ± 20.08 mg/kg on the 4^th^ day. By comparing the aroma profile characteristics related to the VFCs, it was found that the scores of the floral fragrance, fruity fragrance, honey fragrance, nutty fragrance, and creamy fragrance increased with the fermentation time of the CFRW, while the scores of the rice fragrance showed the opposite trend (Figure 7).

**Table 2:** Concentrations of the volatile flavor compounds in the coffee flower rice wine during the fermentation (mg/kg).

CAS	VFCs	Internal standards	F0	F1	F2	F3	F4
78–83-1	2-methyl-1-propanol	3-Methyl-1-butyl-1,1-d2 alcohol	147.60 ± 20.33^e^	332.54 ± 3.02^d^	406.45 ± 6.12^c^	439.99 ± 7.88^b^	470.96 ± 8.53^a^
123–51-3	3-methyl-1-butanol	3-Methyl-1-butyl-1,1-d2 alcohol	1349.72 ± 163.33^e^	2072.60 ± 67.51^d^	2539.94 ± 29.07^c^	3003.97 ± 19.22^b^	3152.65 ± 80.73^a^
111–27-3	1-Hexanol	n-Hexyl-1,1-d2 alcohol	136.64 ± 50.56^e^	617.86 ± 26.78^d^	1192.53 ± 67.58^b^	859.51 ± 7.91^c^	1406.52 ± 74.85^a^
106–24-1	Geraniol	(±)-Linalool-d3	637.35 ± 79.30^d^	757.71 ± 65.05^c^	3691.17 ± 297.81^b^	4179.53 ± 95.98^a^	3496.98 ± 153.48^b^
127–91-3	β-Pinene	(±)-Linalool-d3	81.58 ± 7.69^d^	111.63 ± 1.15^c^	549.28 ± 15.90^b^	715.35 ± 18.81^a^	567.65 ± 30.79^b^
5,989-27-5	D-Limonene	(±)-Linalool-d3	40.74 ± 2.23^c^	39.51 ± 4.74^c^	196.10 ± 22.30^b^	219.16 ± 22.48^a^	189.53 ± 13.14^b^
13,466–78-9	3-Carene	(±)-Linalool-d3	34.74 ± 3.11^e^	44.49 ± 3.98^d^	261.90 ± 26.02^b^	309.25 ± 0.20^a^	247.05 ± 18.25^c^
78–70-6	Linalool	(±)-Linalool-d3	70.25 ± 1.09^e^	66.42 ± 0.83^d^	228.97 ± 18.64^c^	270.59 ± 3.17^a^	253.58 ± 6.18^b^
106–22-9	Citronellol	(±)-Linalool-d3	10.84 ± 0.67^d^	9.67 ± 1.10^d^	131.44 ± 9.46^c^	265.04 ± 7.42^b^	370.67 ± 19.73^a^
141–78-6	Ethyl acetate	Ethyl acetate-d3	272.85 ± 50.15^e^	484.48 ± 49.08^d^	796.99 ± 31.52^c^	1449.69 ± 89.50^b^	2151.73 ± 241.39^a^
123–66-0	Ethyl hexanoate	Ethyl hexanoate-d11	6.85 ± 0.64^e^	9.07 ± 1.16^d^	47.75 ± 1.25^c^	41.57 ± 7.35^b^	67.73 ± 7.16^a^
106–30-9	Ethyl heptanoate	Ethyl octanoate-d15	0.18 ± 0.02^e^	0.53 ± 0.07^d^	5.99 ± 0.47^c^	8.75 ± 0.13^b^	12.20 ± 0.64^a^
106–32–1	Ethyl octanoate	Ethyl octanoate-d15	0.00 ± 0.00^e^	2.92 ± 0.37^d^	32.74 ± 3.17^c^	49.58 ± 0.20^b^	73.15 ± 4.08^a^
123–29-5	Ethyl nonanoate	Ethyl octanoate-d15	0.00 ± 0.00^d^	1.45 ± 0.13^c^	12.94 ± 1.37^b^	21.83 ± 0.10^a^	21.64 ± 1.65^a^
110–38-3	Ethyl decanoate	Ethyl octanoate-d15	0.62 ± 0.14^e^	4.85 ± 0.27^d^	34.35 ± 4.19^c^	52.37 ± 1.35^b^	73.36 ± 5.70^a^
106–33-2	Ethyl dodecanoate	Ethyl octanoate-d15	0.00 ± 0.00^e^	3.18 ± 0.06^d^	18.45 ± 2.20^c^	25.96 ± 0.52^b^	38.81 ± 4.03^a^
124–06-1	Ethyl tetradecanoate	Ethyl octanoate-d15	2.38 ± 0.68^e^	21.30 ± 0.26^d^	116.77 ± ±15.56^c^	158.74 ± 4.13^b^	222.64 ± 25.31^a^
628–97-7	Ethyl hexadecanoate	Ethyl octanoate-d15	8.34 ± 6.73^e^	25.74 ± 0.18 ^d^	158.81 ± 23.36^c^	228.55 ± 1.17^b^	320.16 ± 30.61^a^
544–35-4	Ethyl linoleate	Ethyl octanoate-d15	0.00 ± 0.00^e^	2.87 ± 0.62^d^	20.85 ± 13.47^c^	25.87 ± 2.23^b^	38.96 ± 9.42^a^
100–52-7	Benzaldehyde	2-Phenylethyl acetate-d3	2.25 ± 0.25^e^	1.73 ± 0.14^d^	5.91 ± 0.04^b^	6.43 ± 0.19^a^	5.53 ± 0.24^c^
100–51-6	Benzyl alcohol	2-Phenylethyl acetate-d3	3.93 ± 0.97^e^	6.32 ± 0.31^d^	66.42 ± 6.66^c^	83.00 ± 0.72^a^	73.20 ± 3.67^b^
60–12-8	Phenylethyl alcohol	2-Phenylethyl acetate-d3	5.04 ± 0.21^e^	28.67 ± 2.25^d^	153.50 ± 14.64^c^	367.37 ± 13.64^b^	405.40 ± 20.08^a^
3,777-69-3	2-Pentyl-furan	Ethyl octanoate-d15	8.92 ± 0.65^c^	5.71 ± 1.45^d^	23.61 ± 1.88^b^	31.72 ± 1.90^a^	28.27 ± 2.66^a^

Different lowercase superscripts indicate significant differences among comparisons of the same row of data (*p* < 0.05).

As shown in Table 3, there was a marked discrepancy in the contribution of the VFCs to the overall flavor of the rice wine fermented for different days (*p* < 0.05). The OAVs of most VFCs were more than 1 at the initial stage of the fermentation (F1), except for ethyl dodecanoate and ethyl linoleate. Among them, the OAV was significantly increased for the majority of the compounds compared with the unfermented samples (*p* < 0.05). This suggested that the 21 VFCs could contribute to the overall flavor of the CFRW only during the period of F1. Notably, ethyl octanoate was first detected in the samples of F1 compared to the unfermented samples. Moreover, this ester made a significant contribution to the flavor of the CFRW. However, the contribution of these compounds to the flavor of the CFRW varied significantly as the fermentation progressed (*p* < 0.05). Further analysis elucidated that five VFCs with a higher OAV were regarded as the most potentially characteristic flavor profiles of the CFRW, which included linalool, geraniol, ethyl acetate, 1-hexanol, and 3-methyl-1-butanol. They endowed the rice wine with a desirable fragrance. For example, linalool added a lemon aroma to the rice wine, while 1-hexanol had a fat fragrance. Of these, linalool was responsible for the highest contribution to the flavor of the CFRW, followed by geraniol. Although the CFRW contained a lower benzyl alcohol and 2-pentyl-furan content, both of them still contributed significantly to the flavor of the CFRW due to their lower detection thresholds, particularly in the later fermentation stage (*p* < 0.05).

**Table 3:** Odor activity value of the volatile flavor compounds in the coffee flower rice wine.

VFCs	Odor threshold (mg/Kg)	F0	F1	F2	F3	F4	Odor description
2-methyl-1-propanol	6.506	22.69 ± 3.12^e^	51.11 ± 0.46^d^	62.47 ± 0.94^c^	67.63 ± 1.21^b^	72.39 ± 1.31^a^	Nailpolish-like fragrance
3-methyl-1-butanol	0.040	33743.00 ± 4083.25^e^	51815.00 ± 1687.75^d^	63498.50 ± 726.75^c^	75099.25 ± 480.50^b^	78816.25 ± 2018.25^a^	Apple fragrance
1-Hexanol	0.006	22773.33 ± 8426.67^e^	102976.67 ± 4463.33^d^	198755.00 ± 11263.33^b^	143251.67 ± 1318.33^c^	234420.00 ± 12475.00^a^	Fat fragrance
Geraniol	0.007	91050.00 ± 11328.57^d^	108244.29 ± 9292.86^c^	527310.00 ± 42544.29^b^	597075.71 ± 13711.43^a^	499568.57 ± 21925.71^b^	Rose fragrance
β-Pinene	1.500	54.39 ± 5.13^e^	74.42 ± 0.77^d^	366.19 ± 10.60^c^	476.90 ± 12.54^a^	378.43 ± 20.53^b^	Resinous fragrance
D-Limonene	1.000	40.74 ± 2.23^c^	39.51 ± 4.74^c^	196.10 ± 22.30^ab^	219.16 ± 22.48^a^	189.53 ± 13.14^b^	Citric, orange, and berry fragrance
3-Carene	9.300	3.74 ± 0.33^d^	4.78 ± 0.43^c^	28.16 ± 2.80^b^	33.25 ± 0.02^a^	26.56 ± 1.96^b^	Resinous fragrance
Linalool	2.2 × 10^−4^	319318.18 ± 4954.55^c^	301909.09 ± 3772.73^c^	1040772.73 ± 84727.27^b^	1229954.55 ± 14409.09^a^	1152636.36 ± 28090.91^b^	Lemon fragrance
Citronellol	0.062	174.84 ± 10.81^d^	155.97 ± 17.74^d^	2120.00 ± 152.58^c^	4274.84 ± 119.68^b^	5978.55 ± 318.23^a^	Rose fragrance
Ethyl acetate	0.005	54570.00 ± 10030.00^e^	96896.00 ± 9816.00^d^	159398.00 ± 6304.00^c^	289938.00 ± 17900.00^b^	430346.00 ± 48278.00^a^	Fruity fragrance
Ethyl hexanoate	0.005	1370.00 ± 128.00^d^	1814.00 ± 232.00^c^	9550.00 ± 250.00^b^	8314.00 ± 1470.00^b^	13546.00 ± 1432.00^a^	Fruity fragrance
Ethyl heptanoate	1.9 × 10^−3^	94.74 ± 10.53^e^	278.95 ± 36.84^d^	3152.63 ± 247.37^c^	4605.26 ± 68.42^b^	6421.05 ± 336.84^a^	Pineapple fragrance
Ethyl octanoate	0.013	0.00 ± 0.00^e^	224.62 ± 28.46^d^	2518.46 ± 243.85^c^	3813.85 ± 15.38^b^	5626.92 ± 313.85^a^	Brandy fragrance
Ethyl nonanoate	1.200	0.00 ± 0.00^d^	1.21 ± 0.11^c^	10.78 ± 1.14^b^	18.19 ± 0.08^a^	18.03 ± 1.38^a^	Grape fragrance
Ethyl decanoate	1.500	0.41 ± 0.02^e^	3.23 ± 0.18^d^	22.90 ± 2.79^c^	34.91 ± 0.90^b^	48.91 ± 3.80^a^	Coconut fragrance
Ethyl dodecanoate	5.900	0.00 ± 0.00^e^	0.54 ± 0.01^d^	3.13 ± 0.37^c^	4.40 ± 0.09^b^	6.58 ± 0.68^a^	Floral fragrance
Ethyl tetradecanoate	4.000	0.60 ± 0.17^e^	5.33 ± 0.07^d^	29.19 ± 3.89^c^	39.69 ± 1.03^b^	55.66 ± 6.33^a^	Beeswax-like fragrance
Ethyl hexadecanoate	2.000	4.17 ± 3.37^e^	12.87 ± 0.09^d^	79.41 ± 11.68^c^	114.28 ± 0.59^b^	160.08 ± 15.31^a^	Creamy fragrance
Ethyl linoleate	4.500	0.00 ± 0.00^e^	0.64 ± 0.14^d^	4.63 ± 2.99^c^	5.75 ± 0.50^b^	8.66 ± 2.09^a^	Fresh fragrance
Benzaldehyde	0.350	6.43 ± 0.71^e^	4.94 ± 0.40^d^	16.89 ± 0.11^b^	18.37 ± 0.54^a^	15.80 ± 0.69^c^	Cherry and almond fragrance
Benzyl alcohol	0.020	196.50 ± 48.50^d^	316.00 ± 15.50^c^	3321.00 ± 333.00^b^	4150.00 ± 36.00^a^	3660.00 ± 183.50^b^	Cherry and almond fragrance
Phenyl ethanol	0.564	8.94 ± 0.37^e^	50.83 ± 3.99^d^	272.16 ± 25.96^c^	651.37 ± 24.18^b^	718.79 ± 35.60^a^	Rose fragrance
2-Pentyl-furan	0.006	1486.67 ± 108.33^c^	951.67 ± 241.67^d^	3935.00 ± 313.33^b^	5286.67 ± 316.67^a^	4711.67 ± 443.33^a^	Fruity fragrance

The flavor characteristic descriptions are sourced from https://pubchem.ncbi.nlm.nih.gov and https://www.chemicalbook.com. Different lowercase superscripts indicate significant differences among comparisons of the same row of data (*p* < 0.05).

### 3.4 Relationship between the microorganisms and VFCs in the CFRW

**Figure 8:**
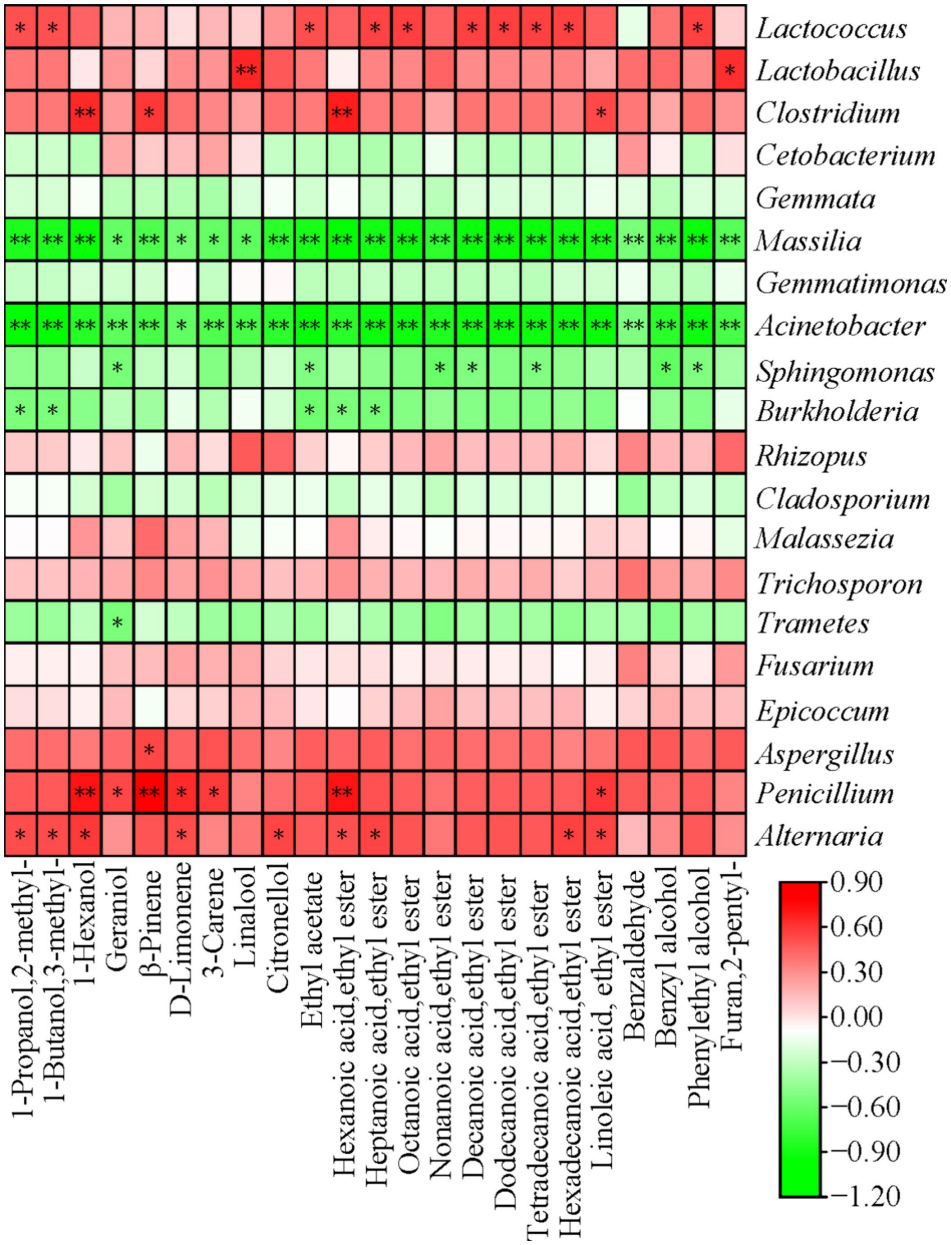
Correlation analysis between the dominant genera and volatile metabolites in the CFRW. ^*^p < 0.05 and ^**^p < 0.01; red and green mean positive and negative correlations, respectively.

The analysis of the correlation between the microbial community and the VFCs of the CFRW at the genus level was shown in Figure 8. It was observed that 11 microbial genera were highly correlated with the 23 VFCs of the CFRW (|*ρ*| > 0.6, *p* < 0.05). The results showed that there were 16 significant positive correlations and 23 significant negative correlations between the microbial genera and VFCs (|*ρ*| > 0.6, *p* < 0.05). Among them, *Acinetobacter* established extremely significant negative correlations with nearly all VFCs (|ρ| > 0.6, *p* < 0.01). *Massilia* and *Sphingomonas* were significantly negatively correlated with geraniol, but *Penicillium* showed the inverse correlation. In this study, *Sphingomonas* had significant negative correlations with seven VFCs (|ρ| > 0.6, *p* < 0.05), including geraniol, ethyl acetate, ethyl nonanoate, ethyl decanoate, ethyl tetradecanoate, benzyl alcohol, and phenylethyl alcohol. *Burkholderia* was significantly negatively correlated with five VFCs, 2-methyl-1-propanol, 3-methyl-1-butanol, ethyl acetate, ethyl hexanoate, and ethyl heptanoate (|ρ| > 0.6, *p* < 0.05). *Lactococcus* established significant correlations with three alcohols and seven esters, which indicated that the major microbial genera participated in the synthesis of these VFCs (|ρ| > 0.6, *p* < 0.05). In the fermentation process of the CFRW, *Lactobacillus* was significantly positively correlated to 2-pentyl-furan and linalool (|ρ| > 0.6, *p* < 0.05). Meanwhile, *Clostridium* had similar correlations with *β*-pinene, ethyl linoleate, 1-hexanol, and ethyl hexanoate (|ρ| > 0.6, *p* < 0.05).

In this research, there were 12 significant positive correlations and one significant negative correlation between the fungal genera and VFCs (|ρ| > 0.6, *p* < 0.05). Among them, *Aspergillus* only had a significant positive correlation with β-pinene (|ρ| > 0.6, *p* < 0.05). *Penicillium* was significantly correlated to D-limonene, 3-carene, ethyl linoleate, 1-hexanol, β-pinene, and ethyl hexanoate (|ρ| > 0.6, *p* < 0.05). In this study, significant positive correlations were found between *Alternaria* and 2-methyl-1-propanol, 3-methyl-1-butanol, 1-hexanol, D-limonene, citronellol, ethyl hexanoate, ethyl heptanoate, ethyl hexadecanoate, and ethyl linoleate.

## 4 Discussion

Aroma and flavor are the most important indicators for determining the quality of rice wine and affect consumer preference (Mao et al., 2023), which are mainly formed by the starting crude and complex microbial metabolism during the brewing process. The interactions of these flavor compounds determine the final flavor characteristics of rice wine. VFCs that are associated with the flavor of rice wine have been frequently determined and identified by researchers, such as alcohols, esters, phenols, and aldehydes (Lu et al., 2017; Huang et al., 2018; Jiang et al., 2020; Liang et al., 2020; Chen et al., 2021; Qian et al., 2023). Interestingly, variations in VFCs may lead to changes in the microbial community and vice versa (Huang et al., 2018).

Alcohols are widespread and important VFCs in wine, which can improve the mellow sensory quality and the body of wine (Fan et al., 2011). It has been reported that the optimal level of higher alcohols (below 300 mg/kg) can produce a fruity aroma and make a fuller flavor of wine; they are acceptable if the concentration is between 300 and 400 mg/kg, but strong irritation might be formed if the content exceeds 400 mg/kg (Luan et al., 2018). In this study, compared with the unfermented samples of the CFRW (F0), the content of the higher alcohols (2-methyl-1-propanol, isoamyl alcohol, and 1-hexanol) significantly increased and exceeded 400 mg/kg in the trial groups, especially in the late stage of the fermentation. Interestingly, there were three higher alcohols (linalool, citronellol, and phenylethyl alcohol) with concentrations below 400 mg/kg, except for phenylethyl alcohol fermented for 4 days. 2-Methyl-1-propanol and isoamyl alcohol are key aroma compounds that make a great contribution to the flavor of Chinese rice wine (Koay et al., 2022). Thus, isoamyl alcohol is usually considered to be one of the main higher alcohols in wine, which provides an alcoholic aroma and largely provides a spicy taste to rice wine (Yang et al., 2024). Beyond that, it may generate bitterness when its concentration is more than the appropriate level (Yu et al., 2022). Furthermore, a lower level of 1-hexanol endows rice wine with a fruity aroma and slight bitterness (Losada et al., 2012). Phenylethyl alcohol endows rice wine with a rose-honey flavor and a sweet taste, which can mask the alcohol smell, increase the intensity of the caramel-like aroma, and reduce the intensity of the fruity aroma of rice wine (Pires et al., 2014; Yu et al., 2019). Therefore, these findings indicate that the latter three higher alcohols may play an important role in neutralizing the negative effect on the CFRW flavor caused by a higher level of 2-methyl-1-propanol, isoamyl alcohol, and 1-hexanol. Integrating our understanding of the interactions of VFCs, it was concluded that perhaps these higher alcohols improved the complexity of the CFRW flavor in this study.

Interestingly, coffee flowers contain VFCs, such as phenyl ethanol and geraniol. Moreover, it was found that geraniol had an extreme OAV level in the fermented samples of the CFRW, particularly on the 3^rd^ day. As we all know, it is one of the most widely natural spices with a mild, rose smell and a bitter taste. The result showed that geraniol detected in the CFRW was very likely derived from coffee flowers, and it contributed vital flavors to the CFRW. It has been proven that benzaldehyde with an almond fragrance is highly related to the full body of rice wine, and it can produce bitterness if the concentration is too high (Yu et al., 2020; Shen et al., 2021). In the study, the concentration of benzaldehyde was very low (< 6.43 mg/kg) and had a lower level of the OAV in all samples, so it can be considered that it could mainly impart the fullness of rice wine.

Esters are the most abundant flavor substances in rice wine, contributing a pleasant fruit and floral fragrance even at lower concentrations (Fan and Qia, 2006; Chen et al., 2013; Qian et al., 2019). In this study, a total of 10 esters were identified, wherein four esters were found to contribute the most to the flavor of the CFRW, which included ethyl acetate, ethyl hexanoate, ethyl heptanoate, and ethyl octanoate. Some studies reported that ethyl acetate could make rice wine present a fruity and brandy aroma; however, it was still considered to be bitter in terms of sensory evaluation (Koay et al., 2022). Ethyl hexanoate, which is often considered sweet, has been frequently documented in rice wine, and its aroma is similar to that of apples, bananas, strawberries, and pineapples (Peinado et al., 2006). Ethyl octanoate and 2-pentyl-furan were also reported to have a fruity aroma (Mao et al., 2023). In this study, the scores of the fruity fragrance, honey fragrance, and creamy fragrance increased as the fermentation of the CFRW progressed, according to the analysis of the aroma profile characteristics related to VFCs. Therefore, the fruit flavor of the CFRW was probably related to these four esters mentioned above and 2-pentyl-furan. In addition, among all VFCs detected in this study, ethyl hexadecanoate was the only flavor substance with a creamy fragrance. Hence, it should be considered that these esters and furan could play a promising and important role in improving the quality and flavor of CFRW in future research.

The flavor components of rice wine are affected by microbial diversity. In the study, five VFCs, linalool, geraniol, ethyl acetate, 1-hexanol, and 3-methyl-1-butanol, contributed vital flavors to the CFRW, and they were all significantly negatively correlated with the changes of *Massilia* and *Acinetobacter* with extremely low proportions (|*ρ*| > 0.6, *p* < 0.05). The major microorganisms detected in the CFRW fermented for 4 days in this study were *Lactococcus*, *Lactobacillus*, *Rhizopus,* and *Cladosporium*. According to reports, *Lactococcus* and *Lactobacillus* are of great significance to the flavor formation of rice wine because they can convert carbohydrates, proteins, and lipids of foodstuffs into aroma compounds (Yang et al., 2024). A previous study proposed that *Lactobacillus* may be involved in the synthesis of isoamyl alcohol (Zhang et al., 2017). However, this finding was discrepant with the results of our research. In the fermented samples, a significant positive correlation was found between the relative abundance of *Lactococcus* and the contents of 10 VFCs in the CFRW, such as isoamyl alcohol, ethyl acetate, ethyl octanoate, and phenyl ethanol (|ρ| > 0.6, *p* < 0.05). The result illustrated that the production of these aroma compound profiles was significantly influenced by *Lactococcus*. In the fermentation process of the CFRW, *Lactobacillus* was significantly positively related to 2-pentyl-furan and linalool (|ρ| > 0.6, *p* < 0.05). Meanwhile, *Clostridium* had a positive correlation with 1-hexanol and ethyl hexanoate (|ρ| > 0.6, *p* < 0.05). These results showed that the two microorganisms could be involved in the synthesis of VFCs with a fruit fragrance.

Previous studies have shown that *Rhizopus* was highly related to many flavor compounds of rice wine (Mao et al., 2023). However, there was no significant correlation between *Rhizopus* and the VFCs, which may have been caused by different strains. Recent research reported that *Acinetobacter* was conducive to the formation of a sweet taste in rice wine (Yang et al., 2024). In this study, the relative abundance of *Acinetobacter* in the CFRW was below 1.00% and decreased by less than 0.01% as the fermentation progressed. It implies that increasing the proportion of *Acinetobacter* in future research may shed light on how to balance the bitterness of CFRW with be rich in higher alcohols. It is worth noting that isoamyl alcohol had a significant negative correlation with *Massilia*, *Acinetobacter,* and *Burkholderia*, but a reverse trend was found for *Lactococcus* and *Alternaria*. Moreover, all the five strains mentioned above promoted the synthesis of isoamyl alcohol in the CFRW in this research. Thus, focus should be on the interactions among microorganisms to effectively reduce the production of isoamyl alcohol, thereby improving the quality of CFRW in the future.

## 5 Conclusion

This study explored the dynamic changes of microbial communities with the extension of the fermentation process and their impact on the VFCs of CFRW. The results showed that *Lactococcus* and *Lactobacillus* were key microbial groups, which were responsible for the flavor formation of the CFRW and thus had a great influence on the final quality of the CFRW. In addition, some microorganisms that represented lower abundance in the CFRW contributed significantly to the flavor formation of the rice wine. In this study, 5 VFCs with the highest OAVs, linalool, geraniol, ethyl acetate, 1-hexanol, and 3-methyl-1-butanol, endowed the CFRW with a pleasant aroma of fruit and fat. Therefore, they were regarded as the most potentially characteristic flavor compounds of the CFRW. Our findings can provide ideas for further developing novel products of rice wine and enhancing the quality of CFRW for promoting the additional value of coffee flowers.

## Data Availability

The datasets presented in this study can be found in online repositories. The names of the repository/repositories and accession number(s) can be found in the article/Supplementary material.
