# Supplementary material for: Interaction and dynamic changes of microbial communities and volatile flavor compounds during the fermentation process of coffee flower rice wine
Source: Front Microbiol. 2024 Sep 19;15:1476091. doi: 10.3389/fmicb.2024.1476091 (PMC11446889; doi:10.3389/fmicb.2024.1476091)
Supplement: Supplementary file 1 [file Data_Sheet_1.docx]

Supplementary Material


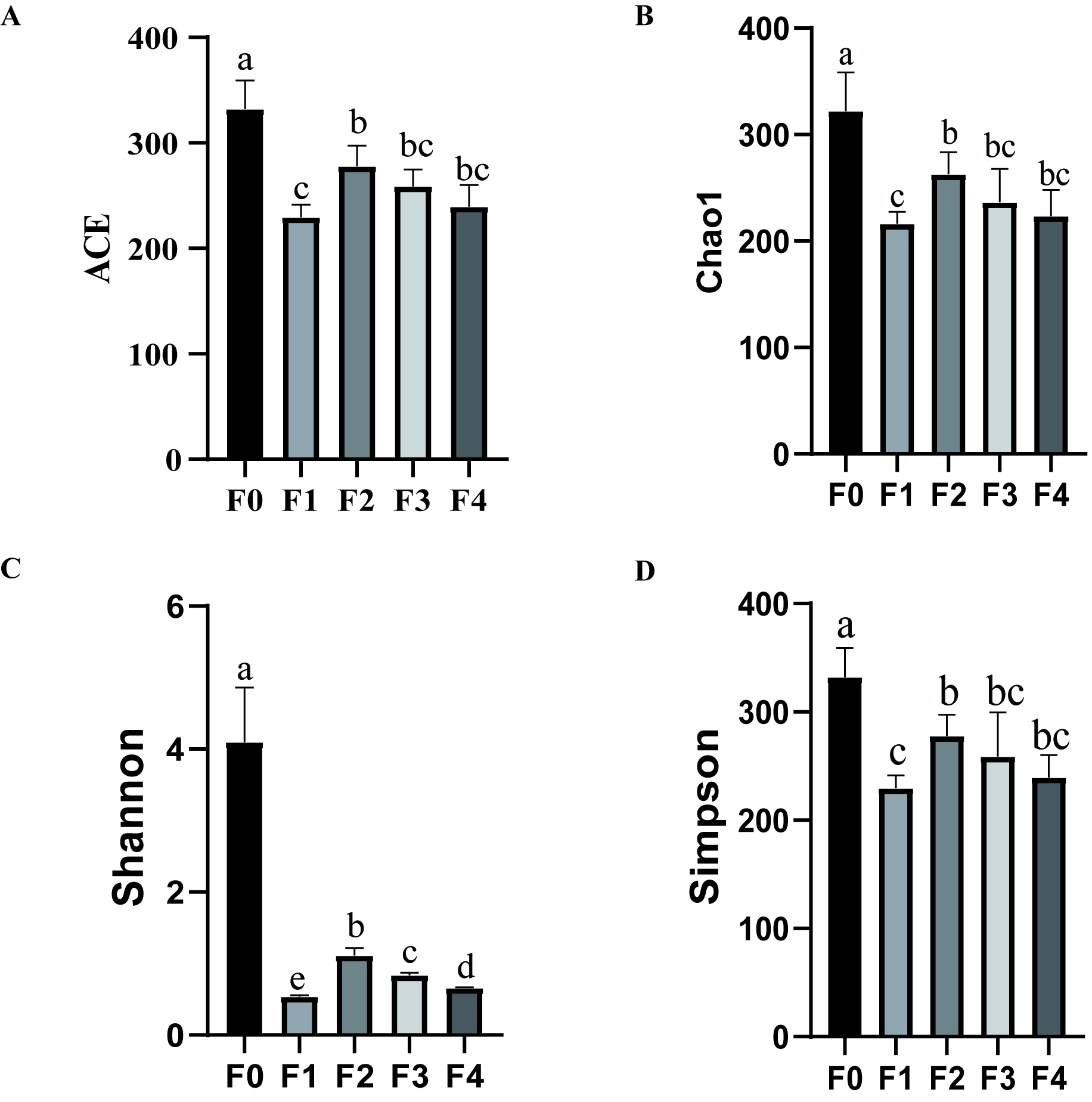


**Supplementary Figure 1.** Comparative analysis of α-diversity indexes of bacterial flora in CFRW at different fermentation stages.


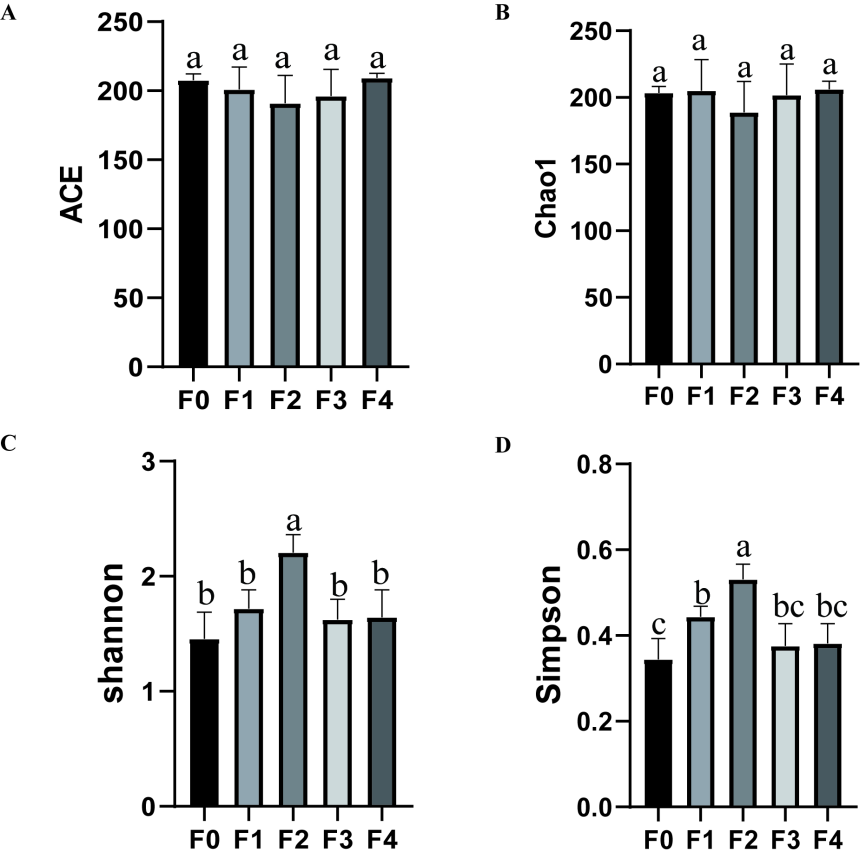


**Supplementary Figure 2.** Comparative analysis of α-diversity indexes of fungal flora in CFRW at different fermentation stages.
